# Bioavailability of Vitamin B_12_ from Dairy Products Using a Pig Model

**DOI:** 10.3390/nu10091134

**Published:** 2018-08-21

**Authors:** Danyel Bueno Dalto, Isabelle Audet, Christiane L. Girard, Jean-Jacques Matte

**Affiliations:** Sherbrooke Research and Development Centre, Agriculture and Agri-Food Canada, Sherbrooke, QC J1M 0C8, Canada; danyel.buenodalto@agr.gc.ca (D.B.D.); isabelle.audet@agr.gc.ca (I.A.); christiane.girard@agr.gc.ca (C.L.G.)

**Keywords:** bioavailability, dairy, pig model, vitamin B_12_

## Abstract

The present study compares the bioavailability of vitamin B_12_ (B_12_) of dairy products or synthetic B_12_, using the pig as an experimental model for humans. Eleven pigs were used in a cross-over design to assess the net portal drained viscera (PDV) flux of blood plasma B_12_ after ingestion of tofu (TF; devoid of B_12_), Swiss cheese (SC), Cheddar cheese (CC), yogurt (YG), and synthetic B_12_ (TB_12_; TF supplemented with cyanocobalamin), providing a total of 25 µg of B_12_ each. PDV blood plasma flow for SC and CC were higher than for TF and TB_12_ (*p* ≤ 0.04) whereas YG was higher than TF (*p* = 0.05). Porto-arterial difference of blood plasma B_12_ concentrations were higher for CC and TB_12_ than for TF and YG (*p* ≤ 0.04) but not different from SC (*p* ≥ 0.15). Net PDV flux of B_12_ was only different from zero for CC. However, the net PDV flux of B_12_ for CC was not different from SC or TB_12_. Cumulative net PDV flux of B_12_ for SC, TB_12_, and CC were 2.9, 4.4, and 8.3 µg 23 h post-meal, corresponding to a bioavailability of 11.6%, 17.5%, and 33.0%, respectively. In conclusion, CC had the best bioavailability of B_12_ among the tested dairy products or compared to synthetic B_12_.

## 1. Introduction

Animal products and by-products are the only natural source of vitamin B_12_ (B_12_) in human diets. Considering that B_12_ is synthesized exclusively by bacteria and archaebacteria (when cobalt is not limiting), ruminant animals (e.g., cows) obtain the vitamin from synthesis by their ruminal microflora. The vitamin is further absorbed and stored in their body, which explain why the tissues and milk of these animals are especially rich in B_12_. 

Among animal-derived products, milk stands out as an excellent source of B_12_. Milk intake was reported to be better correlated with B_12_ status than eggs, red meat, poultry, fish and seafood consumption [1,2]. Using a food-frequency questionnaire, Vogiatzoglou et al. [3] showed that at similar intakes, dairy products have a greater impact on plasma concentrations of B_12_ than the above mentioned products, suggesting a better bioavailability of this vitamin from dairy products. Using a direct measurement, Matte et al. [4] reported greater bioavailability of B_12_ from milk than from the synthetic form (cyanocobalamin) present in most supplements. Considering that similar forms of B_12_ (adenosylcobalamin, hydroxocobalamin, and methylcobalamin) are found in cow’s milk and dairy products [5], they would be expected to have similar B_12_-related nutritional characteristics. However, because of distinct manufacturing processes, the various dairy products are nutritionally different among them or compared to milk. For example, whereas only 60–70% of the original content of B_12_ from milk remains in the curd for Cheddar cheese [6], Swiss cheese increases its original B_12_ content due to the indispensable use of *Propionibacterium shermanii* bacteria, which is known to synthesise this vitamin [7]. For yogurt, the addition of starter cultures does not affect B_12_ concentrations but fermentation of heat-treated milk resulted in losses of 25% [8]. Therefore, it is possible that these different processes impact some nutritional aspects related to this vitamin, such as its bioavailability. 

The present study compares the net flux of B_12_ across portal-drained viscera (PDV) after ingestion of different dairy products or cyanocobalamin (synthetic B_12_) using, as Matte et al. [4], the pig as an experimental model for humans. It aims to determine if, as previously observed for milk, the provision of B_12_ brought by dairy products is better absorbed through the gastrointestinal tract than that of the synthetic form used in vitamin supplements. 

## 2. Methods

The experimental procedures followed the guidelines of the Canadian Council on Animal Care [9] and were approved by the Institutional Animal Care Committee (#490) of the Sherbrooke Research and Development Centre (Sherbrooke, QC, Canada). All animals were cared for according to the recommended code of practice of the National Farm Animal Care Council [10].

## 3. Initial Analysis and Selection of Dairy Products

Because of the wide variety of dairy products available on the market, those commonly consumed worldwide were initially chosen (cheese and yogurt). Considering the huge variation in B_12_ content among these products, different types and brands of cheese and yogurt were selected and analyzed for their content in B_12_. Based on these analyses, Swiss cheese (SC; 32 ng B_12_/g; Agropur, Longueil, QC, Canada), Cheddar cheese (CC; 15 ng B_12_/g; Laiterie de Coaticook, Coaticook, QC, Canada), and plain natural yogurt (YG; 4 ng B_12_/g; Liberté, St-Hubert, QC, Canada) were chosen. Tofu (TF; Horium, Montreal, QC, Canada) was chosen as a negative control diet because foodstuffs from plant origin are naturally devoid of B_12_ [11]. TF was also used as a carrier for the synthetic form of B_12_, cyanocobalamin (TB_12_; positive control). 

In order to minimize variations in B_12_ levels among products throughout the experiment, one single batch of each product was purchased. The concentration of B_12_ in each product was determined (Table 1) before being frozen at −20 °C. One single solution of cyanocobalamin (V-2876, Sigma-Aldrich, St Louis, MO, USA) was prepared, analyzed for its B_12_ content (0.125 mg B_12_/mL) and frozen at −20 °C in individual portions. 

## 4. Preliminary Animal Trial

Considering that pigs are not normally fed this type of foodstuffs, a preliminary animal trial was necessary to assess the maximum consumption of each dairy product in order to standardize B_12_ ingestion among treatments. 

Twenty Yorkshire-Landrace x Duroc pigs were selected at 44.4 ± 4.8 kg of body weight (BW) and 70–77 days of age. They were penned individually (1 m × 1.8 m) and randomly allocated to one of 4 treatments: (1) increasing amounts of TF; (2) increasing amounts of SC; (3) increasing amounts of CC; and (4) increasing amounts of YG. Animals were allowed one single daily meal. When the consumption of the tested products did not provide the equivalent of 1200 g of dry matter, the meal was complemented with a conventional growing-phase feed after the 1 h feeding trial. Evaluations of maximum ingestion capacity, meal duration, and intestinal health (presence of diarrhea) were performed. The trial ended when a similar average consumption was achieved during two consecutive days. This happened at day 7 for TF, day 8 for SC and CC, and day 10 for YG. In average, pigs were able to eat 2.3 kg of TF, 1.8 kg of SC, 1.8 kg of CC, and 7.2 kg of YG within 1 h. No diarrhea was observed during this preliminary trial.

## 5. Description of Treatments

Based on the results of the B_12_ analyses and the preliminary trial, the experimental dose of B_12_ to be administered to animals was fixed at 25 µg. This amount was found to be sufficient to produce a detectable response of post-prandial portal net fluxes in a previous experiment [12]. It also corresponds to the current daily allowance given to market pigs of this age [13]. Therefore, the total volume of each product to be used was 1670 g of CC, 780 g of SC, 3650 g of YG, 2000 g of TF and 2000 g of TB_12_ (a dice of TF was infused with 200 µL of the cyanocobalamin solution at 0.125 mg B_12_/mL). For SC, considering that 780 g represents less than 70% of a normal pig feed intake, 1 kg of TF was added to the treatment (offered after SC was completely consumed). For YG, based on the above described analysis and trial, it would not be possible to reach the fixed dose of 25 µg by using fresh YG. Therefore, a part of experimental YG was lyophilized (dosed B_12_ concentration was 27.16 ng/g) to be further incorporated into fresh YG just before feeding to animals. Pre-trial analyses of B_12_ concentration were performed in mixes of fresh + lyophilized YG to ensure an accurate concentration.

## 6. Experimental Animals and Palatability Test

Forty four pigs were selected (based on BW and average daily gain) two weeks before surgery and fed ad libitum a conventional growing-phase diet. Diet composition was 87.43% of dry matter, 3243 Kcal of metabolizable energy, 16.4% of crude protein, 3.29% of fat, 2.69% of crude fiber, 0.83% of calcium, and 0.54% of phosphorus. In order to identify pigs with the greatest predisposition to ingest the studied products, a palatability test was performed. Without any fasting period, pigs were offered each product as follows: Day 1 and 2: 400 and 500 g of TF; day 3 and 4: 400 and 500 g of SC; day 5 and 6: 400 and 500 g of CC; day 7 and 8: 750 and 1500 g of YG. The amount of product left in the feeder after 1h was weighted and used to calculate intake. Twenty-six pigs with the highest average intake for all or most of the products were pre-selected for surgery. 

## 7. Surgery

Average BW at surgery was 47.7 ± 7.5 kg. The surgical procedure has been described by Hooda et al. [14]. Briefly, a catheter was inserted in the portal vein at approximately 2.5 cm before its entry into the liver and an ultrasonic flow probe (Transonic Systems, Inc., Ithaca, NY, USA) was installed around the portal vein 1.0 cm distal to the catheter. Another catheter was inserted through the carotid artery up to the junction between the carotid and subclavian arteries. 

Improvements were made on the original pre-, intra-, and post-surgical procedures. Instead of completely withdrawing feed 16h prior to surgery, pigs had access to a total of 400 g of feed overnight. After surgery, pigs had access to 750 g of plain yogurt immediately after waking-up in order to stimulate food consumption. For animals that did not eat during the first morning after surgery, another 750 g of plain yogurt was offered. These procedures reduced fasting time and attenuated the risk of post-operatory gastric ulcerations without any impact on intra- or post-operatory procedures. The portal catheter, originally inserted in direction of the blood flow after installing a V-shaped suture heading the liver, was inserted against the blood flow with the V-shaped suture heading in the opposite direction to the liver. This procedure has reduced the obstruction of catheters by fibrin as compared to previous studies of this laboratory using this technique. The flow probe, which was originally installed after a major dissection of the portal vein, was installed with a minor dissection laterally to the vein. In most cases, the removal of a lymph node that is attached between the vein and the pancreas was necessary. This procedure has reduced surgical time, the risk of rupture of the vein and the occurrence of post-operatory intestinal adhesions without any disturbance of flow probe’s signal.

After these improvements, a total of 15 surgeries were performed; 2 animals were eliminated because of post-operatory intestinal adhesions and 2 animals had their portal catheters blocked.

## 8. Post-Operatory Procedures and Experimental Days

After surgery, animals were penned individually (1 m × 1.8 m) and fed the conventional growing-phase diet described above in a single daily meal, according to their BW (1.0 kg/day until 50 kg BW; 1.2 kg/day from 50–60 kg BW; and 1.5 kg/day after 60 kg BW). Seven to 10 days after surgery, when animals have fully recovered (appetite and normal growth rate), they were gradually adapted (3–5 days) to the metabolic cage (with free access to water). On days -3 and -2 prior to each experimental day (day 0), pigs were adapted to consume an increasing amount of the respective experimental product (1.0 and 2.0 kg for TF; 0.5 and 1.0 kg for SC, 1.0 and 2.0 kg for CC; 1.7 and 3.5 kg for YG). On day -1, no adaptation was performed. On experimental days (one per week), animals were placed in metabolic cages and fed tofu (absent in B_12_) or one of the experimental products providing a total of 25 µg of B_12_. Treatments (TF, SC, CC, YG, and TB_12_) were distributed according to a duplicate 5 × 5 Latin Square design.

Blood samples (4 mL) were collected simultaneously from the two catheters 5 min before the experimental meal and every 60 min post-meal during 23 h Portal blood flow was recorded continuously during 23 h using a flowmeter (Transonic^®^ 400-series; Ithaca, NY, USA) and the PowerLab System (AD Instruments, Colorado Springs, CO, USA). Between experimental days, animals were moved back to their respective pens and fed the basal diet described above.

## 9. Sample Handling and Analyses

Immediately after sampling, arterial and PDV blood were transferred from syringes into EDTA-treated tubes (Vacutainer, Becton Dickinson, Franklin Lakes, NJ, USA). Packed cell volume (PCV) was measured in duplicate on fresh PDV blood by micro-centrifugation. Aliquots of arterial and PDV blood were frozen for hemoglobin determination according to the method of Drabkin [15]. Arterial and PDV plasma were collected after centrifugation of blood at 1800 × *g* for 10 min at 4 °C and frozen at −20 °C for further analysis. Arterial and PDV plasma concentrations of B_12_ were measured in duplicate by radioassay (SimulTRAC-S Radioasssay kit, Vitamin B_12_ (^57^Co)/Folate (^125^I), MP Biomedicals, Diagnostics Division, Orangeburg, NY, USA). For each sample, analyses of plasma B_12_ were made in duplicate. The upper limit for coefficients of variation between duplicate was fixed at ≤4%.

## 10. Calculations and Statistical Analysis

Two animals were not equipped with flow probes (technical reasons) whereas one animal lost flow probe functionality during one profile (YG) and another one during three profiles (SC, CC, and YG). For these animals, the estimation of PDV blood flow was performed using the average blood flows of all other pigs within the same treatment, at each sampling time. The estimated values for these periods were not included in the statistical analysis of PDV plasma flow but were used for the calculation of net PDV flux of B_12_. Net flux of B_12_ across PDV was calculated as described by Girard et al. [16]. Positive net PDV flux indicates release of B_12_ from PDV, whereas negative net PDV flux indicates B_12_ uptake by the PDV. Statistical analyses of arterial concentrations of B_12_, PCV on PDV blood, PDV plasma flows, porto-arterial difference, and net PDV flux of B_12_ were conducted on values for each sampling time. 

All variables were analyzed using the MIXED procedure of SAS (SAS Institute Inc., Cary, NC, USA) [17] according to a cross-over design in which pigs, periods, and treatments were included in the model along with repeated measures in time (equally spaced). When the treatment effect was significant, multiple comparisons between treatments were performed using a *t*-test. Differences were considered significant at *p* ≤ 0.05 and tendencies at 0.05 < *p* ≤ 0.10.

## 11. Results 

Arterial concentrations of B_12_ were not affected by dietary treatments (*p* = 0.18; Table 2) but a time effect was observed in which values gradually decreased throughout the 23 h profile period (181.7 ± 7.3 to 162.1 ± 7.3 pg/mL; *p* < 0.001). Although an interaction treatment × time was observed (*p* = 0.03), no specific pattern could be associated to any particular treatment.

Packed cell volume in the portal blood was affected by dietary treatments (*p* = 0.01). Values for TF and TB_12_ (33.5 ± 0.4 and 33.8 ± 0.5%) were or tended to be higher than SC, CC, and YG (31.9 ± 0.4, 32.5 ± 0.4, and 32.4 ± 0.4%, respectively; *p* ≤ 0.07). A time effect (*p* < 0.001) was observed in which PCV gradually decreased during the first 11 post-prandial hours (from 35.6 ± 0.7 to 31.5 ± 0.7%) but remained stable thereafter until the end of the profile period (32.2 ± 0.7). No interaction treatment × time was observed (*p* = 0.14).

Portal-drained viscera plasma flow was affected by dietary treatments (*p* = 0.01). Values for SC and CC were higher (*p* ≤ 0.04) than TF and TB_12_ whereas YG was higher than TF (*p* = 0.05; Table 2). A time effect (*p* < 0.001) was observed, with maximal values reached at the first post-prandial hour (1.30 ± 0.04 vs 1.09 ± 0.04 L/min for the pre-prandial PDV plasma flow) and this was followed by a gradual decrease until the end of the sampling period (1.14 ± 0.04 L/min). No treatment × time interaction was observed (*p* = 0.19) on this variable.

Porto-arterial differences of plasma B_12_ concentrations were affected by dietary treatments (*p* = 0.03). Values differed from zero (*p* ≤ 0.01) only for CC and TB_12_ and were higher than for TF and YG (*p* ≤ 0.04) but not different from SC (*p* ≥ 0.15; Table 2). No time effect or interaction treatment × time were observed (*p* ≥ 0.39). Net PDV flux of B_12_ tended to be affected by dietary treatments (*p* = 0.06). Values differed from zero (*p* ≤ 0.01) only for CC. Although the average net PDV fluxes of B_12_ (per min) for SC and TB_12_ were not statistically different from zero (*p* ≥ 0.15), their cumulative net PDV flux for the whole 23 h post-meal were numerically positive at 2.9 and 4.4 µg, respectively, corresponding to a calculated bioavailability of 11.6 and 17.5%. Net PDV flux of B_12_ (per min) for CC was higher than that of TF and YG (*p* ≤ 0.02) but not different from SC and TB_12_ (*p* ≥ 0.13; Table 2). Cumulative net PDV flux of B_12_ for CC during the whole post-prandial period of 23 h was 8.3 µg, corresponding to a calculated bioavailability of 33.0%.

## 12. Discussion

To the best of our knowledge, the present study using a net PDV flux approach to assess the amount of vitamin B_12_ absorbed from dairy products through the gastrointestinal tract is unique in scientific literature. Pigs were used because this species is recognized as a reliable and valuable experimental model for studies in human nutrition [18]. More specifically for B_12_, this is supported by previous results of this laboratory [12] reporting that several aspects of the nutritional metabolism of B_12_ in pigs are similar to that in humans [19,20]. 

Although dietary treatment effects on PCV were unexpected, the values for all treatments were within the normal range. Therefore, the most likely explanation for the lower PCV in SC, CC, and YG compared to TF and TB_12_ would be hemodilution of PDV blood. The calculated total provision of B_12_, dry matter, and protein were standardized among treatments, however, levels of sodium were higher for SC, CC, and YG compared to TF (Table 1). Dietary salt intake is known to alter extracellular fluid volume [21]. Pigs in SC, CC, and YG treatments consumed between 16.6 and 41.8 times more salt than in TF, representing 125.8 to 316.3% of the daily amount of salt normally fed to 70–125 kg pigs, and this was ingested in one single meal. This high acute salt consumption might have caused a higher flow of extracellular fluid at the PDV level and increased the plasma fraction of portal blood. Incidentally, portal plasma flow of SC, CC, and YG were also greater than in TF. As for sodium, calculated total provisions of fat were not standardized between treatments and were higher for SC, CC, and YG compared to TF (Table 1). Fat has long been known to impact intestinal venous blood flow [22]. Indeed, Chou and Coatney [23], after evaluating the impact of various nutrients on postprandial intestinal hyperemia concluded that micellar fatty acids were the most effective in increasing intestinal blood flow. In this sense, SC, CC, and YG provided between 1.5 and 5.5 times more fat than TF. 

The mean porto-arterial difference and net PDV flux of B_12_ following a meal not supplemented with B_12_ (TF) (Table 2) were not different from zero, as previously observed by Matte et al. [4,12]. These same authors reported conflicting results for cyanocobalamin (equivalent to TB_12_ in the present study). Matte et al. [12] supplemented pigs with levels of 25 or 250 µg of cyanocobalamin in corn starch + casein-based diets and observed net B_12_ flux different from zero whereas in Matte et al. [4] the net flux of that same synthetic form of B_12_ did not differ from zero in diets incorporating B_12_ solutions in plant-based feedstuff (44 and 71 µg B_12_). Such inconsistent results may be related to the known effect of different food matrixes on B_12_ absorption [24]. In dairy cows, Artegoitia et al. [25] reported a better absorption of B_12_ after a post-ruminal infusion of a solution of cyanocobalamin + casein hydrolysate than after infusions of cyanocobalamin + whey protein or free cyanocobalamin solutions. Proteins are known to slow gastric emptying [26] and, although both casein and whey are proteins with high B_12_-binding capacity [27], casein (hydrolyzed or not) has a gastric emptying time 33% slower than whey [28]. This may be caused by formation of curd-like structures by caseins once in the stomach whereas whey remains liquid. Therefore, it appears that the food matrix effect on the absorption of vitamin B_12_ would be related to the rate of the vitamin release from this food matrix leaving the stomach. In this sense, a gradual gastric release of B_12_ would enhance the duration and efficiency of its absorption whereas the presence of bulky transient arrival of B_12_ at the site of absorption would result in a greater amount of unabsorbed B_12_ in the intestinal lumen because B_12_ receptors in the ileum are saturable [29]. This hypothesis is in line with the fact that increasing dietary levels of the vitamin, which is likely related to a greater gastric release, decreases the efficiency of B_12_ absorption [30]. 

Mean porto-arterial difference and net PDV flux of B_12_ for YG were not different from zero (Table 2). Considering its similarity with milk (high protein liquids) one would expect YG absorption to be comparable to that of milk (8–10%) [4]. Compared to milk, yogurt is richer in proteins (milk enriched with milk solids) which, in addition to various buffers produced during the fermentation process, provide a greater buffering capacity to this dairy product [31]. According to Jalan et al. [32], following ingestion of a standard 250 mL dose of yogurt or whole milk, 31 and 10 meq of gastric HCl would be required to reduce the pH of the meals to a pH of 2. In this sense, the present YG treatment (3650 g) would require 8.7 times more HCl than the milk treatment of Matte et al. [4] at 1300 g to reduce the pH of the meals to a pH of 2, which is critical for the release of B_12_ from its binding proteins in milk [33]. Additionally, according to Rioux and Turgeon [34], high viscous food matrixes may impair the digestion of nitrogenous compounds such as casein, the greater binder of B_12_ in milk [33]. In this sense, it has to be stated that the present YG treatment (fresh + lyophilized yogurt) was much more viscous than milk or even regular yogurt. Considering that the bioavailability of protein-bound B_12_ is dependent on the gastric degradation of B12-binding proteins, the above mentioned factors suggest that the release of cobalamin from these binding proteins, a crucial step in B_12_ intestinal absorption, was impaired.

For cheeses, mean porto-arterial difference and net PDV flux of B_12_ for SC did not differ from zero whereas CC did (Table 2). However, it has to be stated that net PDV flux of B_12_ for CC was not statistically different from that of SC. In fact, the cumulative net PDV flux of B_12_ for SC was numerically positive and corresponded to a calculated bioavailability of 11.6%. Although this value is lower than CC (33.0%), it is comparable to values reported by Matte et al. [12] for semi-purified diets supplemented with 25 µg of cyanocobalamin (9.7%) and Matte et al. [4] using milk preparations containing 44 or 71 µg of B_12_ (8–10%). 

**Figure 1:**
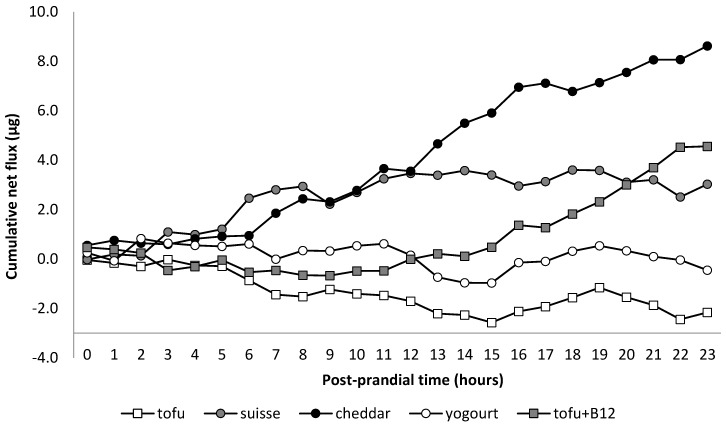
Calculated cumulative net portal-drained viscera flux of B_12_ (µg) by post-prandial time. TF = tofu; SC = Swiss cheese; CC = Cheddar cheese; YG = yogurt; TB_12_ = tofu + vitamin B_12_.

Although most steps of the manufacturing process of SC and CC are similar, differences in starter cultures, in particular the use of Propionibacterium shermanii that is known to synthesize B_12_ [11], may explain the higher concentration of B_12_ in SC as compared to CC. In fact, the present SC was twice as concentrated in B_12_ as CC (32 µg/g vs. 15 ug/g). In this sense, it has to be stated that the mass of B_12_-containing foodstuff (SC curd) ingested was half of that of CC (780 g vs. 1670 g). This combination of greater concentration and smaller mass implies that the release of B_12_ was proportionally faster in SC. Another important difference in the manufacturing process of these cheeses is the timing of the salting of the curd. For CC, salt is applied prior to the pressuring procedure, whereas for SC it is done after. Salting the curd stimulates the leak of whey that will be lost during pressuring. The lower loss of whey in SC implies that it is richer in plasmin (protease). In fact, Richardson and Pearce [35] reported that SC has 2–3 times more plasmin than CC. This protease preferentially hydrolyzes casein suggesting that links casein-casein or casein-B_12_ would be weaker in SC than in CC. In fact, those authors indicated that the extent of casein degradation in SC was related to plasmin content, whereas little evidence of plasmin degradation was observed in CC. Together with the more B_12_-concentrated mass in SC, this suggests that the digestion of SC may release B_12_ faster and in a greater amount than CC, saturating B_12_ receptors in the ileum and losing a higher proportion of B_12_ (unabsorbed) downstream in the intestinal lumen (Figure 1). For CC, the more gradual release of B_12_ may have reduced the saturation of the receptors improving the efficiency of B_12_ absorption.

For SC, however, it cannot be ruled out that the greater presence of B_12_-binding proteins from bacterial origin in SC might have also contributed to differences in bioavailability of B_12_ compared to CC.

## 13. Conclusions

Dairy products can be considered as adequate sources of dietary B_12_. Among the tested products, CC had the best bioavailability of B_12_ followed by TB_12_ and SC. This might be related to the rate of release of B_12_ from the food matrix leaving the stomach modulating the saturation of B_12_ receptor in the ileum and the efficiency of intestinal absorption of this vitamin.

Further investigations are needed to assess the importance of this phenomenon for B_12_ bioavailability in foodstuffs. 

## Figures and Tables

**Table 1:** Composition of the experimental products (as-fed basis) and their calculated provision of dry matter, protein, fat, salt, and vitamin B_12_^1^.

Item	Tofu	Swiss Cheese	Cheddar Cheese	Yogurt ^2^
	Composition			
Dry matter, %	34.60	62.40	52.90	12.70 (23.7)
Protein, g/g	0.17	0.27	0.23	0.06 (0.11)
Fat, g/g	0.05	0.27	0.33	0.02 (0.04)
Sodium, mg/g	0.10	5.33	5.00	0.49 (0.91)
Vitamin B_12_, ng/g	0.12	31.88	14.87	3.77 (6.79)
	**Calculated provision per meal**			
Dry matter, g	692.0	833.0	883.0	865.1
Protein, g	340.0	380.6	384.1	401.5
Fat, g	100.0	260.6	551.1	146.0
Sodium, g	0.2	4.26	8.35	3.32
Vitamin B_12_, ng	0.2	25.1	24.8	24.8

^1^ The amount of each experimental product fed was: tofu = 2000 g, Swiss cheese = 780 g, Cheddar cheese = 1670 g, yogurt = 3650 g. ^2^ Values within brackets refer to the preparation of fresh + lyophilized yogurt.

**Table 2:** Average B_12_ arterial concentration, PDV plasma flow, porto-arterial difference, and net PDV flux of vitamin B_12_ during 23 post-prandial hours according to dietary treatments.

Item	Tofu	Swiss Cheese	Cheddar Cheese	Yogurt	Tofu + B_12_	*p* Value
Arterial B_12_, ng/L	173.2 ± 14.2	177.2 ± 13.0	145.4 ± 143.0	187.7 ± 15.2	194.6 ± 16.4	0.18
PDV plasma flow, L/min	0.93 ^c^ ± 0.08	1.31 ^a^ ± 0.08	1.34 ^a^ ± 0.08	1.19 ^ab^ ± 0.09	1.06 ^bc^ ± 0.08	0.01
Porto-arterial difference, ng/L ^1^	−1.36 ^b^ ± 1.56	1.58 ^ab^ ± 1.46	4.68 ^a^ ± 1.53	−0.21 ^b^ ± 1.68	4.78 ^a^ ± 1.81	0.03
Net PDV flux of B_12_, ng/min ^2,3^	−1.50 ^c^ ± 1.84	2.10 ^abc^ ± 1.73	5.99 ^a^ ± 1.81	−0.31 ^bc^ ± 1.98	3.17 ^ab^ ± 2.14	0.06

Different subscribed letters within a row indicate differences between treatments using *t* test (*p* ≤ 0.05). PDV: portal drained viscera. ^1^ Values for tofu, Swiss cheese, and yogurt were not different from zero (*p* ≥ 0.29). ^2^ Values for tofu, Swiss cheese, yogurt, and tofu + B_12_ were not different from zero (*p* ≥ 0.15). ^3^ Because estimated values of PDV plasma flow were not included in the statistical analysis (due to missing data) of PDV plasma flow but were used for the calculation of arterial and PDV flux of B_12_, net PDV flux of B_12_ does not reflect the multiplication of porto-arterial difference by PDV plasma flow.
